# Burnout Among Emergency Medical Technician Students and Practising Professionals in Madrid, Spain: A Cross-Sectional Study on Healthcare Workforce Sustainability

**DOI:** 10.3390/healthcare14101393

**Published:** 2026-05-19

**Authors:** Gregorio Jesús Alcalá-Albert, Gloria Marlén Aldana-de Becerra, Eduardo José Sánchez-Uzcátegui, José Hernández-Ascanio, María Elena Parra-González

**Affiliations:** 1 Department of Clinical and Health Sciences, Loyola University of Andalusia, 14004 Córdoba, Spain; gjalcala@uloyola.es (G.J.A.-A.); ejsanchez@uloyola.es (E.J.S.-U.); 2 CENAMI Research Group, Ministry of National Education of Colombia, 111321 Bogotá, Colombia; galdana415@yahoo.com; 3 Department of Research Methods and Diagnosis in Education, University of Granada, 51001 Ceuta, Spain; elenaparra@ugr.es

**Keywords:** burnout, emergency medical technicians, emergency medical services, occupational health, healthcare workforce, workforce sustainability, prehospital care, students, psychological distress

## Abstract

**Highlights:**

**What are the main findings?**

- High burnout was observed in both EMT students and practising EMT professionals, based on instrument-specific thresholds, with no statistically significant between-group difference detected.
- In the exploratory adjusted model, age was associated with high burnout, whereas professional status and sex were not significant correlates.

**What are the implications of the main findings?**

- The findings may support the inclusion of early psychosocial risk assessment and support strategies during EMT training, particularly before and during clinical placements.
- EMS organisations and educational institutions could use these exploratory findings to inform coordinated approaches to workforce well-being, occupational health, and healthcare workforce sustainability.

**Abstract:**

**Background:** Burnout is a relevant occupational health concern in Emergency Medical Services (EMSs), with potential implications for workforce well-being, occupational health, and the sustainability of prehospital care. Although burnout has been widely studied among healthcare professionals, evidence concerning Emergency Medical Technician (EMT) students remains limited. This exploratory study aimed to estimate high burnout prevalence among EMT students and practising EMT professionals in Madrid, Spain, describe burnout dimensions in both groups, and examine sociodemographic correlates of high burnout status. **Methods:** A cross-sectional comparative study was conducted between March and June 2024 using a convenience sample of 85 participants: 43 EMT students and 42 practising EMT professionals. Burnout was assessed using validated Spanish versions of the Maslach Burnout Inventory: the MBI-SS for students and the MBI-HSS for professionals. Because these instruments are population-specific and rely on different norms and thresholds, between-group comparisons of raw scores were interpreted as exploratory. Descriptive analyses, between-group comparisons with effect sizes, correlation analyses, and an exploratory binary logistic regression model were performed. **Results:** High burnout was identified in 22 EMT students (51.2%) and 23 practising EMT professionals (54.8%), with no statistically significant between-group difference detected (*p* = 0.73; Cramer’s V = 0.04). Between-group comparisons of burnout dimensions showed small effect sizes for Emotional Exhaustion (Cohen’s d = 0.17), Depersonalisation (Cohen’s d = 0.24), and Personal Accomplishment (Cohen’s d = −0.26). Age was positively associated with Emotional Exhaustion (r = 0.29, *p* = 0.008) and Depersonalisation (r = 0.24, *p* = 0.028), and negatively associated with Personal Accomplishment (r = −0.26, *p* = 0.019). In the exploratory adjusted logistic regression model, age was associated with high burnout status (OR = 1.05; 95% CI 1.01–1.10; *p* = 0.017), whereas group and sex were not significant correlates. **Conclusions:** High burnout levels were observed in both EMT students and practising EMT professionals in this regional exploratory sample. However, the findings should be interpreted cautiously due to the cross-sectional design, convenience sampling, modest sample size, limited statistical power, and use of population-specific burnout instruments. These results suggest that burnout-related distress may be relevant across the EMT training-to-practice pathway and support the need for larger longitudinal and multicentre studies incorporating occupational, educational, and organisational variables.

## 1. Introduction

Burnout is a well-documented occupational health concern in healthcare systems and is typically conceptualised through three core dimensions: emotional exhaustion, depersonalisation, and reduced personal accomplishment [1]. Emotional exhaustion refers to feelings of being emotionally overextended and depleted by work demands; depersonalisation involves detached, cynical, or impersonal responses towards service recipients; and reduced personal accomplishment reflects a negative evaluation of one’s competence and effectiveness at work [1,2]. In Emergency Medical Services (EMSs), these dimensions are particularly relevant because professionals are routinely exposed to high-acuity clinical situations, unpredictable work environments, time pressure, shift work, emotionally demanding events, and sustained organisational demands [3,4]. Unlike many hospital-based professionals, EMS workers often provide care in uncontrolled environments, make rapid decisions with limited information, and manage emergencies under operational uncertainty. These characteristics may affect not only worker well-being but also workforce retention, perceived patient safety, quality of care, and the sustainability of prehospital emergency systems [5,6].

Emergency Medical Technicians (EMTs) constitute a central component of the emergency care workforce. Their role involves frontline prehospital care, patient transport, basic life support, coordination with other emergency professionals, and direct contact with patients and relatives in highly stressful circumstances. The occupational environment of EMTs may include repeated exposure to traumatic scenes, critical incidents, death, severe injuries, interpersonal conflict, and emotionally intense encounters. In addition, organisational stressors such as workload, shift schedules, limited recovery time, staffing constraints, and perceived lack of support may contribute to burnout-related distress [3,4,7]. These factors make EMTs a particularly relevant group for occupational health research. However, compared with physicians and nurses working in hospital-based emergency settings, EMTs and other prehospital emergency professionals have received comparatively less attention in the burnout literature [5,8].

Recent evidence has strengthened the relevance of burnout as a specific issue in EMSs and prehospital emergency care. A systematic review focused on EMT and paramedic populations reported that occupational stress and burnout are highly prevalent among these professionals and are associated with physical, emotional, and socio-occupational consequences [9]. The review also highlighted that burnout in EMSs cannot be fully understood by extrapolating findings from hospital-based professionals, because prehospital emergency work involves distinct operational, environmental, and psychosocial demands. Similarly, Kaplan et al. reported that almost 60% of surveyed EMS clinicians were likely to have occupational burnout, reinforcing the need to identify factors associated with burnout in this professional group [10]. Other studies have linked burnout and psychosocial working conditions in EMSs to perceived patient safety, intention to leave the profession, professional role expectations, and broader workforce sustainability concerns [5,6,11,12]. Taken together, this evidence suggests that burnout among EMTs should be addressed as a workforce and healthcare management issue, rather than solely as an individual psychological outcome.

At the same time, burnout-related distress may not be restricted to established professionals. A growing body of research suggests that students in health-related training programmes may also experience emotional exhaustion, depersonalisation or cynicism, and reduced personal accomplishment, particularly when academic demands are combined with clinical exposure, performance pressure, and uncertainty regarding professional competence [13,14]. For students, burnout may emerge in relation to academic workload, evaluation demands, emotional strain, and the transition from classroom-based learning to clinical practice. Although student burnout has been more extensively studied in medical, nursing, and broader health sciences education, the specific situation of EMT students remains less explored. This is important because EMT training may involve early contact with emergency care environments, exposure to real or simulated critical incidents, and the progressive assumption of responsibilities in settings that are emotionally and operationally demanding.

For EMT students, the transition to clinical placements may represent a particularly sensitive period. During this stage, students move from theoretical instruction to supervised participation in emergency care contexts. This transition can involve exposure to patients in acute distress, traumatic scenes, time-sensitive decisions, and emotionally complex interactions with patients, families, and professionals. Although students do not usually carry the same level of responsibility as practising professionals, they may experience uncertainty, fear of error, emotional overload, and difficulty integrating academic expectations with real-world clinical demands. Evidence from prehospital emergency personnel shows that exposure to traumatic scenes, unpredictability, emotional overload, and decision-making pressure are relevant occupational stressors in this field [7]. While this evidence refers mainly to practising professionals, it provides a plausible framework for understanding why burnout-related symptoms may also be relevant during EMT training.

Recent studies in health sciences education further support the need to examine burnout during professional training. Research among nursing students has examined burnout in relation to emotional intelligence, simulation-based learning experiences, and differences between educational pathways [15,16]. Although these studies are not specific to EMT training, they reinforce the broader concern that students exposed to demanding clinical education environments may experience emotional exhaustion, depersonalisation, and reduced personal accomplishment. This literature also suggests that student burnout should be interpreted within the interaction between educational demands, clinical exposure, coping resources, and institutional support. Applying this perspective to EMT students may be especially useful because prehospital training involves early exposure to emergency scenarios and to the organisational culture of EMSs.

Despite the growing relevance of burnout research in EMSs and health sciences education, important gaps remain. First, many studies focus on practising professionals, while comparatively fewer examine students preparing to enter prehospital emergency occupations. Second, studies involving students and professionals are often conducted separately, which limits understanding of how burnout-related distress may be distributed across the training-to-practice pathway. Third, comparative research focusing specifically on EMT students and practising EMT professionals within the same regional context remains scarce. Such comparisons may provide preliminary evidence on whether burnout-related distress is already detectable during training and how it compares with that observed in professional EMS practice. However, this type of comparison requires methodological caution, particularly when different population-specific instruments are used to assess burnout among students and professionals.

A further methodological consideration concerns the assessment of burnout across different populations. In student samples, burnout is commonly assessed using the Maslach Burnout Inventory–Student Survey (MBI-SS), whereas in healthcare professionals, it is commonly assessed using the Maslach Burnout Inventory–Human Services Survey (MBI-HSS). Both instruments are based on the three-dimensional conceptual framework of burnout, but they are not psychometrically interchangeable and rely on different population-specific norms and thresholds. Therefore, direct comparisons of raw scores between students and professionals should be interpreted cautiously. In comparative studies such as the present one, prevalence estimates based on instrument-specific thresholds may provide a more appropriate descriptive approach than assuming full metric equivalence between instruments. This issue is particularly relevant for interpreting between-group differences and for avoiding overstatement of findings.

Understanding burnout across the EMT training-to-practice pathway has potential implications for occupational health, education, and healthcare workforce sustainability. If burnout-related distress is already present during training, preventive strategies may need to begin before full entry into professional practice. Educational institutions may have a role in preparing students for clinical placements, supporting emotional regulation, and identifying early signs of burnout-related distress. EMS organisations, in turn, may need to consider occupational health strategies that address psychosocial risks, workload, shift patterns, exposure to traumatic events, and access to support systems. Nevertheless, given the cross-sectional nature of most available evidence, including the present study, such implications should be understood as practice-oriented considerations rather than evidence of intervention effectiveness.

Therefore, this study aimed to: (1) estimate the prevalence of high burnout among EMT students and practising EMT professionals in Madrid, Spain; (2) describe and compare burnout dimensions between both groups as exploratory analyses; and (3) examine the association between sociodemographic variables, specifically age, sex, and group, and high burnout status. Given the use of population-specific versions of the Maslach Burnout Inventory for students and professionals, between-group comparisons of raw burnout scores were interpreted cautiously. We hypothesised that high burnout would be observed in both groups and that increasing age would be associated with a higher likelihood of high burnout.

## 2. Materials and Methods

### 2.1. Study Design and Setting

This study used an exploratory cross-sectional comparative design to examine burnout among Emergency Medical Technician (EMT) students and practising EMT professionals. Data were collected between March and June 2024 in the Community of Madrid, Spain. The study was conducted in two complementary EMS-related settings: educational settings involving EMT vocational training programmes and professional settings involving practising EMTs working in public emergency medical services.

The study was reported in accordance with the Strengthening the Reporting of Observational Studies in Epidemiology (STROBE) guidelines for cross-sectional studies. Given its cross-sectional design, the study was intended to describe burnout prevalence and explore sociodemographic correlates of high burnout status, without making causal or longitudinal inferences.

### 2.2. Participants and Sampling

The study population comprised two groups associated with Emergency Medical Services (EMSs): second-year Emergency Medical Technician (EMT) students undertaking clinical placements and practising EMT professionals working in public emergency services in the Community of Madrid. Students were eligible if they were enrolled in the second year of an EMT vocational training programme and were undertaking supervised clinical placements at the time of data collection. Practising professionals were eligible if they were certified EMTs actively working in public EMSs during the study period. For both groups, additional inclusion criteria were voluntary participation and provision of written informed consent. Participants with incomplete responses on the burnout instruments or key sociodemographic variables were excluded from the final analytical sample.

A non-probability convenience sampling strategy was used due to feasibility constraints and the difficulty of accessing both EMT students in clinical placement settings and frontline practising EMT professionals during the study period. Recruitment was coordinated through the academic management of participating training centres and EMS supervisors. Because recruitment was mediated through these institutional channels and a complete denominator of all eligible individuals was not systematically recorded, a formal response rate could not be calculated.

The final analytical sample included 85 participants: 43 EMT students assigned to clinical placements in Basic Life Support units and 42 practising EMT professionals. No missing data were observed for the variables included in the final analyses.

The use of convenience sampling may have introduced selection bias, as participants who agreed to take part may differ from non-participants in terms of stress exposure, perceived burnout, motivation to participate, or availability. Therefore, the findings should not be interpreted as representative prevalence estimates for all EMT students or practising EMT professionals in Spain. The participant flow is summarised in Supplementary Figure S1.

### 2.3. Measures

Sociodemographic variables included age, sex, and group status (EMT student or practising EMT professional). Burnout was assessed using population-specific Spanish versions of the Maslach Burnout Inventory. EMT students completed the Maslach Burnout Inventory–Student Survey (MBI-SS), whereas practising EMT professionals completed the Maslach Burnout Inventory–Human Services Survey (MBI-HSS).

Both instruments are based on the three-dimensional conceptualisation of burnout and assess Emotional Exhaustion, Depersonalisation, and Personal Accomplishment. However, the MBI-SS and MBI-HSS are designed for different populations and contexts. The MBI-SS assesses burnout in academic or training settings, whereas the MBI-HSS assesses burnout in human service and healthcare-related occupational settings. Although the two instruments measure conceptually related dimensions, they are not psychometrically interchangeable and rely on different population-specific norms and thresholds.

For this reason, direct comparisons of raw scores between EMT students and practising EMT professionals were considered exploratory and interpreted with caution. The main comparative interpretation was based on instrument-specific classifications of high burnout rather than assuming full metric equivalence between the MBI-SS and MBI-HSS. This approach allowed burnout to be assessed using the version most appropriate for each population while explicitly acknowledging the limits of between-group comparability.

Items in both instruments are rated on a 7-point Likert-type scale ranging from 0 (“never”) to 6 (“every day”), with higher scores on Emotional Exhaustion and Depersonalisation indicating higher burnout, and lower scores on Personal Accomplishment indicating higher burnout.

### 2.4. Operational Definition of High Burnout

For descriptive purposes, burnout dimensions were classified according to established instrument-specific cut-off points. In the professional group, assessed with the MBI-HSS, high-risk scores were defined as Emotional Exhaustion ≥ 27, Depersonalisation ≥ 10, and Personal Accomplishment ≤ 33. In the student group, assessed with the MBI-SS, high-risk scores were defined as Emotional Exhaustion ≥ 15, Depersonalisation ≥ 7, and Personal Accomplishment ≤ 22.

For the prevalence analysis and the binary logistic regression model, a dichotomous high burnout variable was created separately within each cohort. Participants were classified as having high burnout when they met the high-risk threshold in at least one of the three burnout dimensions according to the corresponding instrument-specific cut-off points.

This operational definition was used to provide a descriptive indicator of burnout risk within each population. Because the MBI-SS and MBI-HSS rely on different norms and cut-off values, this binary classification should not be interpreted as evidence of full psychometric equivalence between students and professionals. Rather, it was used as a pragmatic, instrument-specific approach to describe and compare the proportion of participants reaching high-risk burnout thresholds in each group.

### 2.5. Data Collection Procedure

Data were collected through anonymous, in-person questionnaire administration between March and June 2024. For the student cohort, recruitment and data collection were coordinated with the academic management of the participating EMT vocational training centres. For the professional cohort, recruitment and data collection were coordinated with EMS supervisors. In both groups, potential participants received information about the purpose of the study, the voluntary nature of participation, confidentiality of responses, and their right to withdraw before submitting the questionnaire.

Participants who agreed to take part provided written informed consent before completing the questionnaire. Questionnaires were completed individually and anonymously, without the collection of directly identifying personal information. The average completion time was approximately 15 min.

Because recruitment was coordinated through educational and EMS institutional channels, and the total number of eligible individuals who were informed about the study was not systematically recorded, a formal response rate could not be calculated. The final analytical sample included only participants with complete data for sociodemographic variables and burnout measures.

### 2.6. Sample Size and Statistical Power:

No formal a priori sample size calculation was performed because this was an exploratory study and recruitment was constrained by access to EMT students and practising EMT professionals during the study period. The final sample size was determined by the number of eligible participants who agreed to participate and provided complete data.

To contextualise the interpretation of non-significant between-group findings, a statistical power analysis was conducted for a two-tailed independent-samples comparison, assuming 43 EMT students and 42 practising EMT professionals, an alpha level of 0.05, and a conventional medium effect size (Cohen’s d = 0.50). Under these assumptions, the estimated statistical power was 0.625. This indicates that the study had limited power to detect medium effects and was underpowered to detect small between-group differences. Therefore, non-significant results should not be interpreted as evidence of equivalence between students and professionals.

In line with the exploratory nature of the study, the regression model was deliberately restricted to a small number of theoretically relevant sociodemographic variables to reduce the risk of overfitting.

### 2.7. Statistical Analysis

Data analysis was performed using IBM SPSS Statistics, version 29.0 (IBM Corp., Armonk, NY, USA). Descriptive statistics were calculated for all study variables. Categorical variables were summarised using frequencies and percentages, and continuous variables were summarised using means and standard deviations. The distribution of continuous variables was examined using the Shapiro–Wilk test.

The primary descriptive comparison between EMT students and practising EMT professionals was based on the prevalence of high burnout according to instrument-specific thresholds. Differences in high burnout prevalence between groups were examined using the chi-square test, and effect size was reported using Cramer’s V.

Because the MBI-SS and MBI-HSS are population-specific instruments and are not psychometrically interchangeable, between-group comparisons of raw burnout dimension scores were treated as exploratory. These exploratory comparisons were performed using independent-samples t-tests, with effect sizes reported as Cohen’s d. The absence of statistically significant between-group differences was not interpreted as evidence of equivalence.

Associations between age and burnout dimensions were examined using Pearson correlation coefficients in the total sample and separately by group. Differences in burnout dimensions according to sex were assessed using independent-samples t-tests, with effect sizes reported as Cohen’s d.

To examine sociodemographic correlates of high burnout, an exploratory binary logistic regression model was fitted with high burnout status as the dependent variable. Age, group (EMT student vs. practising EMT professional), and sex were included as independent variables based on theoretical relevance and to avoid model overfitting given the modest sample size. The model was considered exploratory because relevant occupational and contextual variables, including years of experience, working hours, shift patterns, workload, case intensity, type of EMS unit, and exposure to traumatic events, were not available. Odds ratios (ORs), 95% confidence intervals (95% CIs), and *p* values were reported.

Multicollinearity among predictors was assessed using variance inflation factors (VIFs). Model calibration was evaluated using the Hosmer–Lemeshow goodness-of-fit test. Statistical significance was set at *p* < 0.05 for all analyses.

### 2.8. Ethical Considerations

The study was conducted in accordance with the ethical principles of the Declaration of Helsinki. Ethical approval was obtained from the Ethics Committee of Universidad Loyola Andalucía. All participants provided written informed consent prior to participation.

## 3. Results

**Figure 1:**
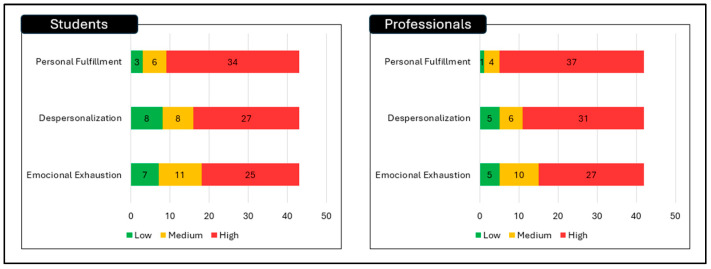
Prevalence of burnout and levels of involvement.

The results are presented in five sections: sample characteristics, burnout dimensions and high burnout prevalence, exploratory between-group comparisons, associations with age and sex, and the exploratory logistic regression model. Descriptive and inferential findings are summarised in Table 1, Table 2, Table 3 and Table 4 and Figure 1.

### 3.1. Sample Characteristics

The final analytical sample included 85 participants, of whom 43 were second-year Emergency Medical Technician (EMT) students and 42 were practising EMT professionals working in public Emergency Medical Services (EMSs) in the Community of Madrid. All participants included in the final analyses had complete data for the sociodemographic variables and burnout measures used in the study.

The two groups differed markedly in age, as expected given their different positions within the EMT training-to-practice pathway. EMT students had a mean age of 20.7 years (SD = 2.4), whereas practising EMT professionals had a mean age of 36.9 years (SD = 8.6). This age difference should be considered when interpreting subsequent analyses, particularly those involving age and group status in the same model. In this sample, age may partly capture differences related to professional experience, cumulative exposure to EMS work, or other unmeasured occupational variables.

Regarding sex distribution, the student group included 21 men (48.8%) and 22 women (51.2%). In the professional group, 26 participants were men (61.9%) and 16 were women (38.1%). The sex distribution therefore showed a higher proportion of men among practising EMT professionals than among EMT students, although this difference was not statistically significant in the descriptive comparison reported in Table 1. These characteristics are summarised in Table 1.

### 3.2. Burnout Dimensions and High Burnout Prevalence

Mean scores for the three burnout dimensions are presented in Table 2. Among EMT students, the mean Emotional Exhaustion score was 28.4 (SD = 10.2), the mean Depersonalisation score was 11.7 (SD = 6.5), and the mean Personal Accomplishment score was 26.3 (SD = 8.9). Among practising EMT professionals, the corresponding mean scores were 30.1 (SD = 9.8) for Emotional Exhaustion, 13.2 (SD = 5.9) for Depersonalisation, and 24.1 (SD = 7.8) for Personal Accomplishment.

Because EMT students completed the MBI-SS and practising EMT professionals completed the MBI-HSS, raw scores should not be interpreted as fully equivalent across groups. Both instruments assess conceptually related dimensions of burnout, but they are population-specific and rely on different norms and thresholds. For this reason, the descriptive presentation of mean scores is provided to characterise each group, whereas between-group comparisons of raw scores are treated as exploratory.

Based on instrument-specific thresholds, high burnout was identified in 22 EMT students, representing 51.2% of the student group, and in 23 practising EMT professionals, representing 54.8% of the professional group. The study did not detect a statistically significant difference in high burnout prevalence between groups (*p* = 0.73). The effect size for this comparison was negligible (Cramer’s V = 0.04), indicating a very small association between group status and high burnout classification in this sample. These findings are summarised in Table 3 and Figure 1.

The absence of a statistically significant difference in high burnout prevalence should not be interpreted as evidence that the two groups are equivalent. Given the modest sample size and the limited statistical power of the study, small or moderate differences between EMT students and practising professionals may not have been detected. Therefore, the prevalence findings should be interpreted descriptively and cautiously, as exploratory evidence that high burnout levels were observed in both cohorts.

### 3.3. Exploratory Between-Group Comparisons of Burnout Dimensions

Exploratory between-group comparisons were conducted for the three burnout dimensions. These analyses were retained because they provide descriptive information on the distribution of burnout dimensions across the two cohorts, but they should be interpreted with caution due to the use of different population-specific MBI versions.

No statistically significant between-group difference was detected for Emotional Exhaustion. EMT students had a mean score of 28.4, whereas practising EMT professionals had a mean score of 30.1. The difference was not statistically significant (*p* = 0.40), and the effect size was small (Cohen’s d = 0.17), indicating only a small difference in the direction of higher Emotional Exhaustion among practising professionals.

For Depersonalisation, EMT students had a mean score of 11.7, whereas practising EMT professionals had a mean score of 13.2. This difference was also not statistically significant (*p* = 0.24). The effect size was small (Cohen’s d = 0.24), again suggesting a small difference in the direction of higher Depersonalisation among practising professionals.

For Personal Accomplishment, EMT students had a mean score of 26.3, whereas practising EMT professionals had a mean score of 24.1. The difference was not statistically significant (*p* = 0.20). The effect size was small and negative (Cohen’s d = −0.26), indicating that students had slightly higher Personal Accomplishment scores than professionals. Because lower Personal Accomplishment is generally interpreted as reflecting higher burnout, this pattern should be interpreted cautiously and descriptively rather than as evidence of a robust between-group difference.

Overall, the exploratory comparisons of burnout dimensions did not detect statistically significant differences between EMT students and practising EMT professionals. However, these non-significant findings should not be interpreted as demonstrating equivalence between groups. The small effect sizes suggest that any observed differences in this sample were limited in magnitude, but the study may not have had sufficient statistical power to detect smaller but potentially meaningful differences.

### 3.4. Associations with Age and Burnout Dimensions

Associations between age and burnout dimensions were examined in the total sample and separately by group. In the total sample, age was positively correlated with Emotional Exhaustion (r = 0.29, *p* = 0.008) and Depersonalisation (r = 0.24, *p* = 0.028). Age was negatively correlated with Personal Accomplishment (r = −0.26, *p* = 0.019). This pattern indicates that, in the overall sample, older participants tended to report higher Emotional Exhaustion, higher Depersonalisation, and lower Personal Accomplishment.

When analyses were stratified by group, the pattern differed between EMT students and practising EMT professionals. Among EMT students, no statistically significant correlations were observed between age and any burnout dimension. Specifically, age was not significantly associated with Emotional Exhaustion (r = 0.18, *p* = 0.25), Depersonalisation (r = 0.12, *p* = 0.44), or Personal Accomplishment (r = −0.20, *p* = 0.20). These non-significant findings should be interpreted cautiously, given the relatively narrow age range and modest size of the student subgroup.

Among practising EMT professionals, age was positively correlated with Emotional Exhaustion (r = 0.31, *p* = 0.045) and negatively correlated with Personal Accomplishment (r = −0.33, *p* = 0.033). The association between age and Depersonalisation in professionals was positive but did not reach statistical significance (r = 0.28, *p* = 0.072). These results suggest that, within the professional subgroup, older age was associated with greater Emotional Exhaustion and lower Personal Accomplishment.

The age-related findings should be interpreted as associations rather than causal relationships. In this study, age may partly reflect cumulative exposure to occupational demands, longer time in professional roles, or other unmeasured work-related factors. Because variables such as years of experience, working hours, shift patterns, workload, case intensity, type of EMS unit, and exposure to traumatic events were not collected, the mechanisms underlying the observed age associations cannot be established.

### 3.5. Associations with Sex

Sex differences in burnout dimensions were also examined. Men showed higher Depersonalisation scores than women (13.4 ± 6.2 vs. 11.1 ± 6.0). This difference reached statistical significance (t(83) = 2.02, *p* = 0.046), with an effect size in the small-to-moderate range (Cohen’s d = 0.44). No statistically significant sex differences were observed for Emotional Exhaustion or Personal Accomplishment.

This result suggests that, in this sample, men reported higher levels of Depersonalisation than women. However, the finding should be interpreted with caution due to the modest sample size, the absence of adjustment for occupational variables, and the exploratory nature of the analysis. In addition, sex was not significantly associated with high burnout status in the adjusted logistic regression model, indicating that the observed difference in Depersonalisation did not translate into a significant adjusted association with the binary high burnout outcome.

### 3.6. Exploratory Logistic Regression Model

An exploratory binary logistic regression model was fitted to examine sociodemographic correlates of high burnout status. The dependent variable was high burnout, defined according to instrument-specific thresholds. The independent variables included age, group status, and sex. The model was intentionally parsimonious because of the modest sample size and the absence of relevant occupational and contextual variables.

Age was the only variable significantly associated with high burnout status in the adjusted model. Each additional year of age was associated with a 5% increase in the odds of high burnout (OR = 1.05; 95% CI 1.01–1.10; *p* = 0.017). Group status was not significantly associated with high burnout after adjustment for age and sex. Sex was also not significantly associated with high burnout in the adjusted model.

These regression findings should be interpreted as exploratory. The model included only sociodemographic variables and did not include key occupational or educational factors commonly associated with burnout, such as years of experience, working hours, shift patterns, workload, case intensity, type of EMS unit, exposure to traumatic events, academic workload, or characteristics of clinical placements. Consequently, the model should not be interpreted as an explanatory model of burnout risk. Rather, it provides an initial description of sociodemographic correlates of high burnout in this regional sample.

The association between age and high burnout should also be interpreted cautiously because age differed substantially between EMT students and practising EMT professionals. In this context, age may partly operate as a proxy for professional experience, cumulative occupational exposure, or other unmeasured work-related factors. Therefore, the observed association between age and high burnout does not establish a causal effect of age itself.

## 4. Discussion

This exploratory cross-sectional study examined burnout among EMT students and practising EMT professionals in Madrid, Spain. The main finding was that high burnout levels were observed in both groups, with 51.2% of EMT students and 54.8% of practising EMT professionals meeting instrument-specific criteria for high burnout. The study did not detect statistically significant differences between groups in high burnout prevalence or in the exploratory comparisons of burnout dimension scores. However, these non-significant findings should not be interpreted as evidence of equivalence between EMT students and practising professionals. Given the modest sample size and limited statistical power, small or moderate between-group differences may not have been detected. Therefore, the findings should be understood as exploratory evidence suggesting that burnout-related distress may be relevant across both training and professional EMS contexts.

A key methodological consideration in interpreting these results is the use of different Maslach Burnout Inventory versions for students and professionals. Both instruments are grounded in the three-dimensional conceptualisation of burnout, including emotional exhaustion, depersonalisation, and reduced personal accomplishment [1,2]. The use of the MBI-SS for EMT students and the MBI-HSS for practising EMT professionals allowed burnout to be assessed using instruments adapted to the educational and occupational contexts of each group [14,17]. Nevertheless, these instruments are not psychometrically interchangeable and rely on different population-specific thresholds. For this reason, comparisons of raw scores between groups were treated as exploratory, and the main descriptive interpretation was based on instrument-specific high burnout classifications. This approach provides useful preliminary information but limits the strength of any direct comparison between students and professionals.

The finding that high burnout was observed in both cohorts is consistent with recent literature indicating that burnout and occupational stress are relevant concerns in prehospital emergency care. A systematic review focused on EMT and paramedic populations reported high levels of occupational stress and burnout, as well as physical, emotional, and socio-occupational consequences associated with these conditions [9]. Similarly, Kaplan et al. reported substantial burnout prevalence among EMS personnel, reinforcing the need to identify factors associated with burnout in this professional group [10]. These findings support the interpretation that burnout in EMSs should be considered a specific workforce and occupational health issue, rather than being inferred only from studies of hospital-based professionals.

The present findings also align with studies showing that psychosocial working conditions in EMSs may be associated with patient safety, occupational strain, and workforce sustainability. Baier et al. reported associations between burnout and safety-related outcomes among EMS workers in Germany [5], while Elsässer et al. found that psychosocial working conditions were associated with perceived patient safety among EMS workers [6]. Other recent studies have highlighted the relevance of exposure to prehospital emergency stressors, burnout among prehospital emergency medical personnel, role identities, intention to leave the profession, and organisational challenges in EMS delivery [7,8,11,12]. Taken together, this literature supports the relevance of examining burnout not only as an individual psychological outcome but also as a potential challenge for the stability and sustainability of prehospital emergency services.

The results also contribute to the more limited literature on burnout during health professional training. Although most existing evidence on student burnout comes from medicine, nursing, and broader health sciences education, research has shown that students may experience burnout-related symptoms when academic demands are combined with clinical exposure, uncertainty, and performance pressure [13,14]. Recent studies in nursing education have also examined burnout in relation to emotional intelligence, simulation-based learning experiences, and differences between educational pathways [15,16]. These studies are not directly equivalent to EMT training, but they support the broader argument that students exposed to demanding clinical education environments may experience burnout-related symptoms. In the context of EMT education, clinical placements may represent an especially demanding period, as students are progressively exposed to prehospital care situations, emotionally intense events, and the operational culture of emergency services.

The absence of statistically significant differences between EMT students and practising professionals requires careful interpretation. One possible explanation is that burnout-related distress may already be present during training, particularly when students begin clinical placements and encounter real emergency care environments. Another possibility is that the study was underpowered to detect smaller between-group differences. The statistical power analysis indicated limited power to detect medium effects and insufficient power to detect small effects. Therefore, the lack of statistically significant differences should be interpreted as a non-detection of differences in this sample, not as evidence that burnout levels are equivalent across the two groups.

Age was associated with high burnout in the exploratory adjusted model and was positively correlated with Emotional Exhaustion and Depersonalisation and negatively correlated with Personal Accomplishment in the total sample. This association should not be interpreted causally. In this study, practising professionals were substantially older than EMT students, and age may partly reflect unmeasured differences in professional experience, cumulative exposure to emergency care demands, work history, workload, working hours, shift patterns, type of EMS unit, or exposure to traumatic events. Previous EMS research has shown that burnout and psychosocial strain may be related to working conditions, safety outcomes, exposure to emergency stressors, organisational context, and professional role expectations [5,6,7,8,11,12]. Because these occupational variables were not collected in the present study, it is not possible to determine whether the association between age and burnout reflects age itself or other correlated occupational exposures. Future studies should include these variables to clarify the mechanisms underlying burnout risk in EMT populations.

The exploratory regression model should therefore be interpreted with caution. It included only age, sex, and group because the sample size was modest and additional occupational variables were not available. Although this parsimonious model was appropriate for reducing the risk of overfitting, it limits explanatory power. Burnout in EMSs is likely to be shaped by a broader set of individual, educational, occupational, and organisational factors, including years of experience, working hours, shift schedules, workload, call intensity, repeated exposure to traumatic incidents, perceived organisational support, and professional role expectations [6,7,8,9,11]. The present model should consequently be viewed as a preliminary sociodemographic analysis rather than a comprehensive explanatory model of burnout.

Sex differences were observed only for Depersonalisation, with men reporting higher scores than women. However, sex was not significantly associated with high burnout status in the adjusted model. This finding suggests that sex-related differences may vary depending on the burnout dimension considered and should not be overinterpreted. The result may reflect differences in emotional distancing, coping strategies, occupational exposure, or role-related factors, but the current data do not allow these mechanisms to be examined. Given the modest sample size and the absence of adjustment for work-related variables, this finding should be interpreted as exploratory and should be examined in larger studies.

From an applied perspective, the results suggest that burnout prevention in EMS should not be restricted to established professionals. If burnout-related distress is detectable during training, educational institutions may need to consider early psychosocial risk assessment, preparation for clinical placements, stress-management resources, and structured opportunities for supervised reflection on emotionally demanding experiences. In professional EMS organisations, occupational health strategies may include periodic psychosocial risk assessment, access to psychological support, monitoring of workload and shift patterns, and organisational measures aimed at reducing exposure to preventable chronic stressors. These considerations are consistent with previous EMS research highlighting the relevance of psychosocial working conditions, safety outcomes, professional role expectations, and workforce sustainability [5,6,9,11,12]. However, these implications should be interpreted as practice-oriented considerations derived from the observed pattern of findings, not as evidence that specific interventions are effective.

The study has several strengths. First, it focuses on a relatively underexplored population within emergency care research: EMT students and practising prehospital emergency professionals. Second, it compares two professionally connected groups within the same regional context, providing preliminary evidence on burnout across the EMT training-to-practice pathway. Third, burnout was assessed using Spanish versions of the Maslach Burnout Inventory adapted to each population [14,17,18]. Finally, the study addresses a topic of practical relevance for healthcare workforce planning, occupational health, and the sustainability of prehospital emergency services [19,20].

This study also has important limitations. First, the cross-sectional design precludes causal or longitudinal inferences regarding the development, persistence, or progression of burnout over time. Second, the use of a non-probability convenience sample and the modest sample size limit the generalisability of the findings and reduce statistical power to detect small or moderate between-group differences. Third, the use of two different population-specific versions of the Maslach Burnout Inventory limits the direct comparability of raw scores between EMT students and practising professionals. Fourth, burnout was assessed using self-report instruments, which may be affected by reporting bias or social desirability bias. Fifth, relevant occupational and educational variables were not collected, including years of experience, working hours, shift patterns, workload, case intensity, type of EMS unit, exposure to traumatic events, academic workload, and characteristics of clinical placements. As a result, age may partly reflect unmeasured differences in professional experience or cumulative occupational exposure. Finally, because the number of invited participants was not systematically recorded, a formal response rate could not be calculated. These limitations indicate that the findings should be interpreted as exploratory and hypothesis-generating.

Future research should prioritise multicentre and longitudinal designs following EMT students from training into professional practice. Such studies should include larger and more representative samples and incorporate occupational, educational, organisational, and psychosocial variables that may influence burnout risk. Future work should also examine outcomes relevant to healthcare systems, including intention to leave the profession, absenteeism, perceived patient safety, job satisfaction, and workforce retention. This would allow a more comprehensive understanding of burnout as a potential challenge for the sustainability of prehospital emergency care.

## 5. Conclusions

This exploratory cross-sectional study found high levels of burnout among both EMT students and practising EMT professionals in Madrid, Spain. The study did not detect statistically significant differences in high burnout prevalence between groups; however, this finding should not be interpreted as evidence of equivalence because of the modest sample size, limited statistical power, convenience sampling, and use of population-specific burnout instruments.

Age was associated with high burnout in the exploratory adjusted model, whereas group and sex were not significant correlates. This association should be interpreted cautiously, as age may partly reflect unmeasured differences in professional experience, cumulative occupational exposure, workload, shift patterns, or other contextual factors that were not assessed in this study.

Overall, these findings suggest that burnout-related distress may be relevant across the EMT training-to-practice pathway, including both clinical placement periods and professional EMS practice. Educational institutions and EMS organisations may consider early psychosocial risk assessment and support strategies to promote workforce well-being. Further multicentre and longitudinal studies with larger samples and more detailed occupational, educational, and organisational variables are needed to confirm these findings and clarify modifiable factors associated with burnout in prehospital emergency care.

## Figures and Tables

**Table 1:** Participant characteristics by group.

Variable	EMT Students (*n* = 43)	Practising EMT Professionals (*n* = 42)
Age, mean (SD)	20.7 (2.4)	36.9 (8.6)
Men, *n* (%)	21 (48.8)	26 (61.9)
Women, *n* (%)	22 (51.2)	16 (38.1)

**Table 2:** Burnout dimensions by group: exploratory between-group comparisons.

Burnout Dimension	Students (*n* = 43), Mean (SD)	Professionals (*n* = 42), Mean (SD)	*p* Value	Cohen’s d
Emotional Exhaustion	28.4 (10.2)	30.1 (9.8)	0.40	0.17
Depersonalisation	11.7 (6.5)	13.2 (5.9)	0.24	0.24
Personal Accomplishment	26.3 (8.9)	24.1 (7.8)	0.20	−0.26

**Table 3:** High burnout prevalence by group.

Variable	EMT Students (*n* = 43)	Practising EMT Professionals (*n* = 42)	*p* Value	Effect Size
High burnout, *n* (%)	22 (51.2)	23 (54.8)	0.73	Cramer’s V = 0.04

**Table 4:** Correlations between age and burnout dimensions by group.

Group	Emotional Exhaustion r (*p*)	Depersonalisation r (*p*)	Personal Accomplishment r (*p*)
Total sample	0.29 (0.008)	0.24 (0.028)	−0.26 (0.019)
EMT students	0.18 (0.25)	0.12 (0.44)	−0.20 (0.20)
Practising EMT professionals	0.31 (0.045)	0.28 (0.072)	−0.33 (0.033)

## Data Availability

The data presented in this study are available on reasonable request from the corresponding author. The data are not publicly available due to privacy and ethical restrictions related to the small size of the study subgroups and the potential risk of indirect participant identification.

## Supplementary Materials

The following supporting information can be downloaded at: https://www.mdpi.com/article/10.3390/healthcare14101393/s1, Figure S1. Participant flow diagram.
