# Supplementary figures and images for: Burnout Among Emergency Medical Technician Students and Practising Professionals in Madrid, Spain: A Cross-Sectional Study on Healthcare Workforce Sustainability
Source: Healthcare (Basel). 2026 May 19;14(10):1393. doi: 10.3390/healthcare14101393 (PMC13206574; doi:10.3390/healthcare14101393)

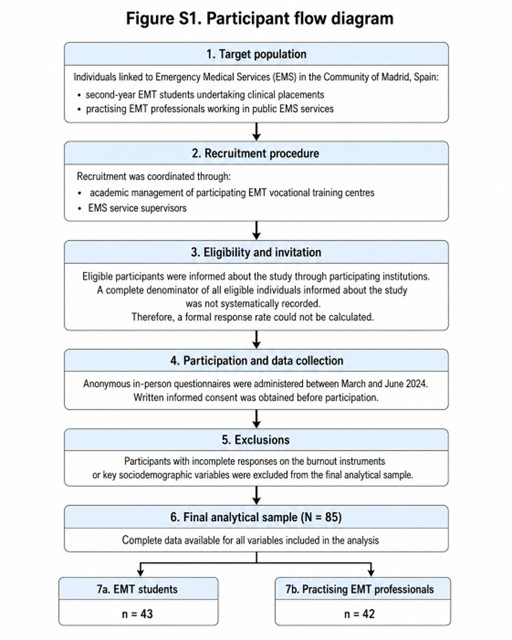

Supplement: Supplementary file 1 [file healthcare-14-01393-s001.zip › healthcare-4310182-supplementary.jpg]
